# Chemical Composition, Antioxidant and Antibacterial Properties of *Achillea collina* Becker ex Heimerl *s.l.* and *A. pannonica* Scheele Essential oils

**DOI:** 10.3390/molecules13092058

**Published:** 2008-09-02

**Authors:** Biljana Bozin, Neda Mimica-Dukic, Mirjana Bogavac, Ljiljana Suvajdzic, Natasa Simin, Isidora Samojlik, Maria Couladis

**Affiliations:** 1 Faculty of Medicine, Department of Pharmacy, Hajduk Veljkova 3, 21 000 Novi Sad, Serbia; E-mail: giba57@eunet.yu (Suvajdzic); 2 Faculty of Sciences, Department of Chemistry, Trg D. Obradovica 3, 21 000 Novi Sad, Serbia; E-mails: mimica@ih.ns.ac.yu (Mimica-Dukic); simin@uns.ns.ac.yu (Simin); 3 Faculty of Medicine, Department of Gynecology and Obstetrics, B. Cosica 37, 21 000 Novi Sad, Serbia; E-mail: mbogavac@yahoo.com (Bogavac); 4 Faculty of Medicine, Department of Pharmacology, Hajduk Veljkova 3, 21 000 Novi Sad, Serbia; E-mail: pstojan@eunet.yu (Samojlik); 5 School of Pharmacy, Department of Pharmacognosy, Panepistimiopolis of Zographou, Zographou 157 71, Athens, Greece; E-mail: kouladi@pharm.uoa.gr (Couladis)

**Keywords:** *Achillea millefolium s.l.*, Essential oil, Chemotypes, Antioxidant, Antimicrobial

## Abstract

The *in vitro* antioxidant and antimicrobial activities of two *Achillea millefolium* (Adanson) Koch *s.l* species essential oils (*A. collina* Becker ex Heimerl *s.l.* and *A. pannonica* Scheele, Asteraceae) originating from the Golija and Radan mountains (Serbia) were investigated. The chemical profiles of the essential oils were evaluated by GC-MS. Antioxidant activity was assessed as free radical scavenging capacity (RSC) towards 2,2-diphenyl-1-picrylhydrazil (DPPH) radicals, together with effects on lipid peroxidation (LP). Antibacterial activity was examined on 21 bacterial strains. Based on the chemical composition of the essential oil, *A. collina s.l.* from Mount Golija was classified as a chamazulene chemotype (tetraploid). The high percentage of oxygenated monoterpenes and absence of azulene in the essential oil obtained from *A. pannonica* from Radan pointing that this population is octaploid. Essential oil of *A. pannonica* expressed stronger antimicrobial activity on almost all tested bacteria. Furthermore, this essential oil expressed higher scavenging effects on DPPH radical (IC_50_ = 0.52 comparing to 0.62 μg/mL). Only in the LP evaluation, essential oil of *A. collina s.l.* from Golija exhibited stronger antioxidant activity (IC_50_ = 0.75 comparing to 2.12 μg/mL).

## Introduction

Yarrow (*Millefolii herba*) is widely used in both folk and official medicine. The main applications of the drug are in the therapy of gastrointestinal complaints, because of its antiphlogistic, spasmolytic, stomachic, carminative and cholagoge effects. Additionally, it is used as a bitter aromatic and to stimulate the secretion of bile [1]. Recent investigations have also pointed out its notable antimicrobial [2,3,4,5] and antioxidant activities [2]. The antimicrobial activity of essential oils in particular has formed the basis of many applications of different aromatic plants [6,7]. This aspect assumes a particular relevance due to an increased resistance of some bacterial strains to the most common antibiotics [8] and antimicrobial agents for food preservation.

Although the biological source of the drug in the Ph. Eur. IV [9] is defined as an *Achillea millefolium* L. *sensu stricto* (*s.s.*) (Asteraceae), most of the phytopharmaceutical books define the source as an *Achillea millefolium* (Adanson) Koch *sensu lato* (*s.l.*) [1,10]. *Achillea millefolium s.l.* represents a cytogenetic and chemically polymorph aggregate of 12 species [11], characterized by a well defined morphological, anatomical and caryological features. However, the existence of the naturally occurring hybrids increases the number of the representatives in the aggregate [12]. The relative ontogenetic stability of the essential oil composition [13,14], quantitative differences in the flavonoid content [15], and the sesquiterpene pattern [16] could be used as an additional tool for the taxonomical differentiation. Furthermore, there is a correlation between the presence of prochamazulene and the chromosome number in the plant [1,12]. As a rule, only the tetraploid plants contain prochamazulene, while most of other caryotypes are azulene-free [14,16]. The oil obtained from the octaploid plants characterize about 80% of oxygenated monoterpenes, and that from the hexaploid species comprised ca. 50% mono- and sesquiterpene hydrocarbons [17].

Although the *Millefolii herba* is widely used in the phytotherapy, the use of this drug is sometimes restricted because allergic contact dermatitis may occur [1,10]. Guaianolide peroxides and sesquiterpene lactones, together with the azulenes in the essential oil of tetraploid plants, are believed to be responsible for itching and inflammatory changes in the skin with the formation of vesicles [1]. Furthermore, the differences in biological activities of the drug could be observed, related to the different compounds present in the plant material used [5,18]. In the present paper the essential oils of two samples of *Millefolii herba*, obtained from *A. collina* Becker ex Heimerl *s.l.* and *A. pannonica* Scheele, two species of *Achillea millefolium* (Adanson) Koch *s.l.* group were chemically characterized. The antioxidant and antibacterial activity of the obtained essential oils were also investigated.

**Table 1:** Chemical composition (%) of *A. collina s.l.* and *A. pannonica* essential oils.

Peak No.	Components	R. I.^a^	*A. collina* (Golija)	*A. pannonica* (Radan)	Identification method^b^
	**Monoterpene Hydrocarbons**		**26.9**	**10.4**	
1	*α-*Thujane	932	0.1	-	RT^∗^ MS
2	*α-*Pinene	935	1.0	2.0	RT GC MS
3	Camphene	951	-	2.0	RT^∗^ MS
4	Sabinene	972	-	2.1	RT^∗^ MS
5	*β*-Pinene	975	22.5	1.1	RT GC MS
6	*β-*Myrcene	987	traces	-	RT^∗^ MS
7	*α-*Phellandrene	1005	traces	-	RT^∗^ MS
8	*α-*Terpinene	1015	2.0	2.1	RT^∗^ MS
10	Z- *β*-Ocimene	1042	traces	-	RT^∗^ MS
11	E- *β*-Ocimene	1052	traces	-	RT^∗^ MS
12	*γ-*Terpinene	1060	1.3	1.1	RT GC MS
	**Oxygenated Monoterpenes**		**20.8**	**78.4**	
9	1,8-Cineole	1034	11.4	40.4	RT GC MS
13	Artemisia ketone	1062	-	4.1	RT^∗^ MS
14	Artemisia alcohol	1083	-	3.1	RT^∗^ MS
15	Linalool	1099	1.0	0.9	RT GC MS
16	Camphor	1145	2.0	11.1	RT GC MS
17	Borneol	1167	-	3.2	RT GC MS
18	Terpinen-4-ol	1176	1.5	4.4	RT GC MS
19	1- *α-*Terpineol	1188	2.6	1.9	RT GC MS
20	Piperitone	1248	-	2.0	RT GC MS
21	E-Chrysanthenyl acetate	1235	-	6.0	RT^∗^ MS
22	Z-Chrysanthenyl acetate	1262	-	1.2	RT^∗^ MS
23	Bornyl acetate	1288	0.1	-	RT GC MS
24	Lavandulyl acetate	1289	2.2	-	RT^∗^ MS
	**Sesquiterpene Hydrocarbons**		**28.0**	**11.1**	
25	E-Caryophyllene	1419	14.9	-	RT GC MS
26	*α-*Humulene	1452	2.0	-	RT GC MS
27	Germacrene D	1490	11.1	11.1	RT^∗^ MS
	**Oxygenated Sesquiterpenes**		**3.0**	**0.00**	
28	Caryophyllene-oxide	1582	3.0	-	RT^∗^ MS
	**Proazulenes**		**19.4**	**0.00**	
29	Chamazulene	1725	19.4	-	RT^∗^ MS
	** Amount of identified compounds**		**98.1**	**99.9**	

^a^ Retention indices relative to C_9_-C_24_ n-alkanes on the HP 5MS column ^b^ RT, comparison with pure standard retention time; GC, gas chromatographic coelution with pure standard; MS, mass spectrometry; RT**^∗^**, comparison of the relative retention time with those obtained from the NIST/NBS, Wiley liberaries spectra and those reported by Adams [23]

## Results and Discussion

### Chemical composition of the essential oils

The amount of the essential oil obtained from *A. collina s.l.* was 0.73 % v/w, and in the one originating from Radan (*A. pannonica*) it was 0.98 % v/w dry matter. The percentage compositions of the both essential oils are presented in Table 1. A total of 22 and 18 chemical constituents, representing 98.44 and 99.96 % of the total content, were identified in each investigated essential oils, respectively.

The oil obtained from the *Achillea collina* *s.l.* collected in Mount Golija was found to be composed of approximately equal amounts of monoterpene (27.19%) and sesquiterpene hydrocarbons (28.02%), followed by oxygenated monoterpenes (20.83%) and proazulenes (19.42%). The main constituents in the essential oil were *β*-pinene (22.52%), chamazulene (19.42%) and E-caryophyllene (14.92%). The presence of chamazulene followed by a high content of *β*-pinene in the essential oil points on tetraploid species [1,12,16]. In the oil originating from Radan (*A. pannonica*) oxygenated monoterpenes (78.52%) were found to be the major class of substances, with the dominance of 1,8-cineole (40.40%) and camphor (11.10%). The chemical composition of the essential oil points on octaploid species [1,12,17].

### Antioxidant activity

The antioxidant activity of two investigated essential oils was evaluated by measuring the scavenging activity on DPPH-radicals and protective effects on LP. In the DPPH-test, the ability of the essential oil to act as the donor of hydrogen atoms or electrons in transformation of DPPH into its reduced form DPPH-H was measured spectrophotometrically. Both assessed essential oils were able to reduce the stable, purple coloured radical DPPH to the yellow-coloured DPPH-H reaching 50 % of reduction with an IC_50_ as follows: 0.62 mg/mL for *A. collina s.l.* and 0.52 mg/mL for *A. pannonica* (Table 2a). Comparison of the RSC of investigated essential oils with those expressed by BHT (0.05 mg/mL) showed that the essential oils expressed stronger scavenging effects than BHT.

**Table 2a:** Neutralisation of DPPH by *A. collina s.l.* and *A. pannonica* essential oils and BHT (as a positive control) in the DPPH assay (results are expressed in percentage of neutralisation).

Source	Concentrations (mg/mL)							
	0.25	0.54	0.88	1.37	2.50	5.00	7.50	IC_50_
*A. collina* (Golija)	17.48	42.90	59.70	70.00	80.60	88.80	90.21	0.62
*A. pannonica* (Radan)	0.00	50.00	66.20	78.00	91.28	97.70	97.99	0.52
BHT (cc in μg/mL)	-	4.62	11.56	23.12	30.11	44.71	55.22	5.37

The identification of essential oil constituents most responsible for the RSC (Table 2b) showed that the most active compounds in the essential oil of *A. collina s.l.* were chamazulene, the mixture of mono- and sesquterpene hydrocarbons and camphor. Also the mixture of mono- and sesquterpene hydrocarbons from the essential oil of *A. pannonica*, together with camphor and some of the monoterpene alcohols, borneol and artemisia alcohol, probably, were the most active scavengers in this oil.

**Table 2b:** DPPH scavenging active compounds identified by TLC dot-blot technique.

Source of essential oil	Compound	Rf values
*A. collina* (Golija)	Camphor	0.73
	Chamazyulene	0.96
	Mixture of mono- and sesquiterpene hydrocarbons	0.98
*A. pannonica* (Radan)	Borneol	0.13
	Camphor	0.73
	Mixture of mono- and sesquiterpene hydrocarbons	0.98

The protective effects of the essential oils in lipid peroxidation have been evaluated by the TBA-assay. Inhibition of LP was determined by measuring the formation of secondary components (malondialdehyde) of the oxidative process, using lecithin liposomes as an oxidizable substrate. With such a model system it is possible to obtain some indications on the real applicability of the oil.

**Figure 1:**
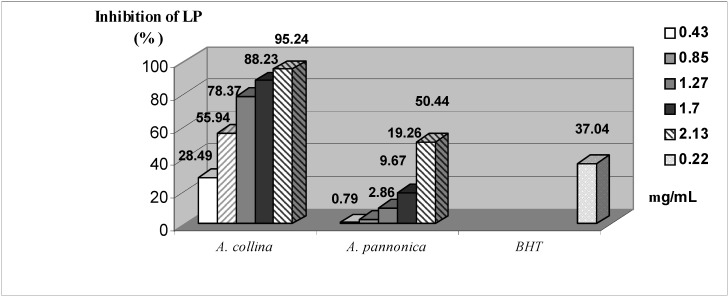
Inhibition of lipid peroxidation (LP) in Fe^2+^/ascorbate system of induction by investigated essential oils and BHT (as a positive control)^a^ in the TBA assay. Concentrations labeled in the legend are expressed in mg/mL.

^a^Essential oils and BHT were diluted in *n-*hexane (a solvent with no antioxidant activity)

Figure 1 shows the antioxidant activity of two investigated essential oils against BHT as a positive control. In general, both examined essential oils expressed similar antioxidant capacity comparing to the BHT and this activity was dose-dependent. This could be confirmed comparing the concentrations of essential oils (0.425 to 2.13 mg/mL) and the stock solution of the BHT (0.22 mg/mL).

The stronger antioxidant activity exhibited by investigated essential oil obtained from octaploid plants (*A. pannonica*), in both the DPPH-test (Table 2a) and TBA-assay (Figure 1), confirms results obtained by DPPH-TLC test (Table 3) showing that some of the oxygenated monoterpenes are mostly responsible for protective effects.

The results gained in both antioxidant assays confirm the previously published data about strong antioxidant effects of the essential oil of *A. millefolium* [2]. They pointed on differences in biological activities, as well, which were related to the different compounds in tested row plant material [5,18].

**Table 3:** Antibacterial activity (inhibition zone expressed in mm)^a^ of two investigated essential oils and antibiotics used as a positive control.

Organism	*A. collina* Golija		*A. pannonica* Radan		Antibiotics^c^	
					*Ampicillin*	*Azitromycin*	
	50%^b^	Pure oil	50%^b^	Pure oil	500 μg/mL	
*Pseudomonas aeruginosa* G-MS	0.0	0.0	0.0	0.0	R	R
*P. aeruginosa* G-MS	0.0	0.0	0.0	0.0	R	R
*P. aeruginosa* ATCC 27853	0.0	0.0	0.0	0.0	R	R
*P. aeruginosa* G	0.0	0.0	0.0	0.0	R	R
*P. aeruginosa* G	0.0	0.0	14.0±0.02	0.0	R	R
*Escherichia coli* ATCC 35218	0.0	0.0	9.0±0.00	9.2.0±0.44	R	R
*E. coli* ATCC 25922	12.0±0.02	14.0±0.02	16.0±0.02	18.6±1.48	R	R
*E. coli* (*haemolytica*) G	0.0	0.0	24.4±0.89	0.0	R	S
*Staphylococcus aureus* G-MS	12.0±0.00	13.1±0.01	40.0±0.00	40.6±1.67	R	R
*S. aureus* G	26.0±0.00	25.8±0.44	62.0±0.00	64.8±0.44	S	S
*S. aureus* G	0.0	20.0±0.01	47.0±0.00	45.8±0.83	S	R
*S. aureus* G	15.0±0.02	15.0±0.00	15.0±0.00	20.2±0.44	S	R
*S. aureus* G	26.0±0.01	34.0±1.22	66.4±0.89	66.6±0.89	S	S
*S aureus* (β-haemolytica) G	8.0±0.00	0.0	43.8±0.83	62.2±1.48	S	R
*Streptococcus pneumoniae* G	16.0±0.01	16.7±0.89	67.0±1.48	65.6±1.82	S	S
*S viridans* G	60.0±0.02	61.8±0.44	60.6±1.82	32.8±0.44	S	S
*S. pyogenes* G	20.0±0.00	21.8±0.83	63.8±0.89	63.6±1.82	S	S
*S. agalactiae* G	18.2±1.48	24.0±0.00	20.0±0.02	25.6±0.55	S	S
*S. agalactiae* G	18.0±0.00	0.0	18.4±0.89	20.2±0.44	S	S
*S. agalactiae* G	0.0	26.4±0.89	15.2±0.44	19.6±1.67	S	S
*S agalactiae* G	16.4±1.14	24.0±0.00	21.0±1.00	24.2±0.44	S	S

^a^ The values represent the average of five determinations ± standard deviations ^b^ Essential oils were diluted in *n*-hexane (the solvent expressed no antibacterial activity) ^c^ The antibiotics’ zone of inhibition mean: R (resistant) < 15 mm, S (sensitive) > 15 mm.

### Antibacterial activity

The antibacterial activity of the investigated essential oils is shown in Table 3. Obtained results revealed that both essential oils exhibited variable levels of antibacterial activity against all tested bacterial strains. According to the literature data [2,19], Gram-positive bacteria seemed to be more sensitive to the examined essential oils than Gram-negative bacteria. Furthermore, the essential oil obtained from *A. collina s.l.* in the most of the cases exhibited stronger antibacterial activity than *A. pannonica* oil (in some of tested *Staphylococcus aureus* and *Streptococcus* strains). This could be due to the presence of high ratio of chamazulene in the essential oil (Table 1).

On the other hand, stronger bacteriostatic activity of *A. pannonica* was observed on *Streptococcus viridans* and one strain of *Streptococcus agalactiae* in comparison to *A. collina* oil (Table 3). This could be explained by notable amounts of 1,8-cineole (40.40%), camphor (11.10%) and borneol (3.22%) in the essential oil. All of the three substances are confirmed as strong antimicrobials on a different range of bacteria [2,20,21].

From the obtained results it is obvious that the chemical composition of the essential oil has important impact on both antioxidant and antimicrobial effects of *Millefolii herba* obtained from different biological sources. The presence of chamazulene increases the antibacterial activity, whereas the antioxidant and scavenging effects of essential oil are related to some other substances, such are camphor and borneol.

## Experimental

### Plant Material

**Figure 2:**
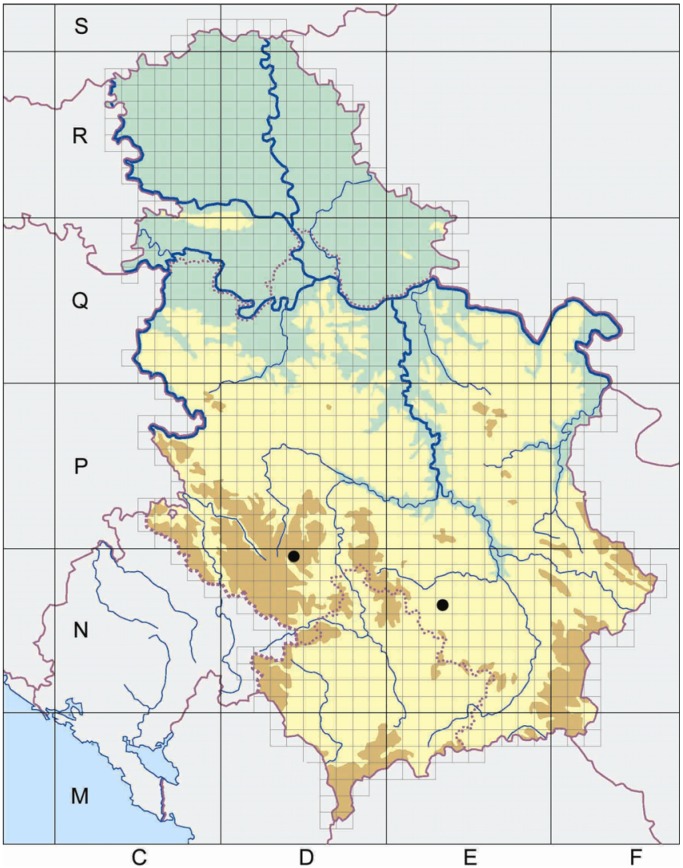
The UTM map of Serbia with labeled localities of plant material collection (Golija-left and Radan-right).

Two commercial samples of yarrow (*Millefolii herba*) together with the complete biological materials were purchased from the local market in Novi Sad, Serbia. One sample (*A. collina* Becker ex Heimerl *s.l.*) originated from Mount Golija (Crni Vrh, UTM 34T DN 1 49) and the second one (*A. pannonica* Scheele) was from Mount Radan (Šopot, UTM 34T EN 1 36), both in Serbia (Figure 2).

Voucher specimens (Ph-Ac No. 22/03-Golija and Ph-Ap No. 23/03-Radan) were confirmed [22] and deposited at the Herbarium of the Laboratory of Pharmacognosy, Department of Pharmacy, Faculty of Medicine, University of Novi Sad.

### Isolation of the Essential Oil

Plant materials were submitted to the hydrodistillation according to Eur. Pharm. 4 [9], using *n*-hexane as a collecting solvent. The oil samples were dried over anhydrous sodium sulfate. The solvent was removed under the vacuum and the quantities of the obtained essential oils were determined gravimetrically.

### Essential Oil Analysis

Qualitative and quantitative analysis of the investigated essential oils were carried out using a Hewlett-Packard 5973-6890 GC-MS system, operating in EI mode at 70 eV, equipped with a split-splitless injector (200 °C) and a flame ionization detector (250 °C). Helium was used as carrier gas (1 mL/min) and the used capillary columns were HP 5MS (30 m x 0.25 mm; film thickness 0.25 μm). The temperature programmes were 60 °C to 280 °C at a rate of 3 °C/min and 60-260 °C at a rate of 3 °C/min, respectively; split ratio, 1:10. Identification of individual compounds was based on coelution and MS analysis. When possible peak relative retention times were compared with those of authentic samples (Carl Roth GmbH; Karlsruhe, Germany). For the other components, mostly sesquiterpenes and aliphatic compounds, for which reference substances were not available, the identification was performed by matching their retention indices and mass spectra with those obtained from authentic samples and/or the NIST/NBS, Wiley spectral libraries and literature data [23].

### Evaluation of Antibacterial Activity

A collection of 21 bacterial strains, including both Gram-positive and Gram-negative bacterial strains, was used. The tested bacteria included three strains of American Type of Culture Collection (ATCC) and 18 strains, including some multiresistant (G-MS), obtained from Clinic of Gynecology and Obstetrics, Clinical Centre of Vojvodina, by direct isolation from patients. For the evaluation of the antibacterial activities of the essential oils, the disk-diffusion method carried out in five repetitions was used [24,19]. The effect of the solvent (*n*-hexane) on the microbial growth was analyzed, too. Furthermore, the effects of two antibiotics, ampicillin and azitromycin were also investigated as positive control.

### Antioxidant Activity

Antioxidant properties of investigated essential oils were evaluated as both free radical scavenging capacity (RSC) and protective effects on the lipid peroxidation (LP).

### Free Radical Scavenging Capacity (RSC)

RSC was evaluated by measuring the scavenging activity of examined essential oils on the DPPH-radicals. The DPPH-assay was performed as described before [19]. Different concentrations of essential oils, ranging from 0.25 to 7.5 mg/mL, were tested. The absorbance of tested and blank control solutions (the same chemicals, except for the sample) was recorded spectrophotometrically at 515 nm one hour after. The *tert*-butylated hydroxytoluene (BHT) was used as a positive control. Four replicates for each sample were recorded. The percentage of RSC was calculated using the following equation:

RSC (%)=100 x (*A_blank_*-*A_sample_* / *A_blank_*)

The IC_50_ value, which represented the concentrations of the essential oil that caused 50% inhibition, was determined by linear regression analysis from the obtained RSC values.

The dot-blot test on TLC silica gel F_254_ aluminium plates stained with the free radical DPPH^°^ was used for fast screening of essential oil compounds responsable for RSC [19]. They were identified comparing the DPPH-TLC chromatogram with control TLC treated with vanillin-sulphuric acid spray reagent.

### Determination of Lipid Peroxidation (LP)

The extent of LP was determined by measuring the color of the adduct produced in the reaction between 2-thiobarbituric acid (TBA) and malondialdehyde (MDA), as an oxidation product in the peroxidation of membrane lipids, by the TBA-assay [19]. The commercial preparation of lecithin liposomes “PRO-LIPO S” pH=5-7 was used as a model-system of biological membranes. The liposomes, 225-250 nm in diameter, were obtained by dissolving the commercial preparation in demineralized water (1:10) in an ultrasonic bath.

Several concentrations of essential oils were tested in this experiment: pure essential oil (2.13 mg/mL), 80, 60, 40 and 20% solution in *n*-hexane (0.425, 0.85, 1.27 and 1.70 mg/mL, respectively). The content of the MDA (TBARS) was determined spectrophotometrically at 532 nm. A control with *n*-hexane was also analyzed. Commercial synthetic antioxidant BHT (0.5 M stock solution, concentration 220.4 μg/mL) was used as a positive control. All reactions were carried out in triplicate.

The percentage of LP inhibition was calculated by the following equation:

I (%) = (A_o_-A_1_)/A_o_ x 100

where A_o_ was the absorbance of the control reaction (complete reaction, without the test compound) and A_1_ was the absorbance in the presence of the inhibitor.
